# Power estimation of tests in log-linear non-uniform association models for ordinal agreement

**DOI:** 10.1186/1471-2288-11-70

**Published:** 2011-05-17

**Authors:** Fabien Valet, Jean-Yves Mary

**Affiliations:** 1 Institut Curie, Ecole des Mines de Paris, INSERM U900, Paris, FRANCE; 2 INSERM U717, Hôpital Saint-Louis, Paris, FRANCE

## Abstract

**Background:**

Log-linear association models have been extensively used to investigate the pattern of agreement between ordinal ratings. In 2007, log-linear non-uniform association models were introduced to estimate, from a cross-classification of two independent raters using an ordinal scale, varying degrees of distinguishability between distant and adjacent categories of the scale.

**Methods:**

In this paper, a simple method based on simulations was proposed to estimate the power of non-uniform association models to detect heterogeneities across distinguishabilities between adjacent categories of an ordinal scale, illustrating some possible scale defects.

**Results:**

Different scenarios of distinguishability patterns were investigated, as well as different scenarios of marginal heterogeneity within rater. For sample size of N = 50, the probabilities of detecting heterogeneities within the tables are lower than .80, whatever the number of categories. In additition, even for large samples, marginal heterogeneities within raters led to a decrease in power estimates.

**Conclusion:**

This paper provided some issues about how many objects had to be classified by two independent observers (or by the same observer at two different times) to be able to detect a given scale structure defect. Our results also highlighted the importance of marginal homogeneity within raters, to ensure optimal power when using non-uniform association models.

## Background

Initially developped in psychometrics to assess the severity of behavioral troubles or disturbances [1-3], ordinal rating scales (ORS) are now essential tools in health research and health care: for example to measure clinical outcomes such as symptom grading [4], pathologists finding [5], disease severity [6], treatment response [7-9], as well as health-related quality of life [10,11]. When the same objects are classified twice on a scale, differences in perception of one observer to another, or of the same observer at two successive times, lead to inter-rater and intra-rater variability. For patients, reproducibility of ratings made using an ORS is a major issue because their classification into one of the different categories may have important consequences on their therapeutic follow-up and possibly on their quality of life. There are two main components of reproducibility. The first component is marginal homogeneity between raters, which corresponds to the differences in raters marginal distributions and refers to the tendencies of a rater to make classifications higher or lower than those of the other rater. The second component is category distinguishability, that is to say the ability for observers to distinguish between categories. Recently, non-uniform association models (NUA) were proposed by Valet *et al. *[12] to estimate degrees of distinguishability between adjacent categories of an ORS. These models allowed to test different patterns of distinguishability and then to give information of the scale structure quality.

When designing a reproducibility study with two observers (or one observer at two different times) assessing the same objects on an ORS, two major questions have to be solved: How many objects has to be classified by the two observers to be able to detect a given heterogeneous pattern of distinguishability between adjacent categories? Is it important to select these objects in an attempt to approximate some marginal distributions? In this study, simulations were used to estimate the power of non-uniform association models to detect heterogeneities across distinguishabilities between adjacent categories as a function of typical distinguishability patterns and total number of objects classified, assuming homogeneous marginal distribution within reader and between readers. Then, for the same numbers of objects classified twice, the influence of different patterns of marginal heterogeneity within reader on power estimate was studied.

## Methods

### Log-linear non-uniform association models

#### Log-linear modelling and parameters interpretation

Classifications of *N *objects by two independent raters *A *and *B *(or by the same rater at two different times) using an ORS with *I *categories can be summarized in a *I *× *I *contingency table. In this table, let us define counts *n_ij _*as the numbers of objects rated *i *(*i *= 1,..., *I*) by observer A and *j *(*j *= 1,..., *I*) by observer B, and suppose that these counts have a full multinomial distribution with expected mean *m_ij _*= *N *× *π*_*ij *_, where *N *is the sample size, and *π_ij _*is a probability distribution on the cells of the *I *× *I *table. Log-linear modelling expresses the logarithm of these *m_ij _*as a linear combination of parameters that illustrates raters effects on categories, as well as sources of agreement and disagreement. For the independence model, which assumes that ratings are statistically independent, the model is written as:(1)

where *μ *is the overall effect and  and  are *A *and *B *effects on category *i *and *j*, respectively. For this model, agreement between raters is expected to be due to chance only.

When analyzing agreement in ordered contingency table, we can usually expect an association between ratings due to the natural ordering of the scale. As described by several authors [12-15], this association between rating is expected to increase as the distance between categories increases. For instance on a five-level severity scale, if an object is rated "1" by *A*, the probability for this object to be rated "5" by *B *is very low [16]. This association can be expressed through odds ratio *τ_ij _*= *m_ii_m_jj_*/*m_ij_m_ji_*. An odds ratio value equal to 1 indicates that the two ratings are independent. From odds ratio *τ*_*ij*_, Darroch and McCloud defined  as the degree of distinguishability (DD) between two categories of an ORS, that is to say the readers' ability to distinguish between these two categories [17]. A DD value close to 1 indicates an almost perfect distinguishability between the two corresponding categories whereas a DD value close to 0 indicates that these two categories are very hard to distinguish.

#### Uniform Association (UA) and Non-Uniform Association (NUA) models

In order to take into account this association, Goodman introduced the uniform association (UA) model. In 2007, Valet *et al. *[12] proposed an equivalent but simpler parameterization of the UA model as:(2)

where *i *= 1,..., *I *and *j *= 1,..., *I*. From the UA model, odds ratio are written as . Hence, DDs between two categories *i *and *j *are written as  assuming that the DDs between categories vary according to the distance between them. However, as pointed out by Valet *et al. *[12] the DDs between adjacent categories are supposed to be constant which can be a limiting *a priori *hypothesis, since it assumes that the categories of the scale are regularly spaced in terms of distinguishabilities; a rather satisfying property for an ORS. They proposed log-linear non-uniform association (NUA) models to take into account the variations of the DDs between both distant and adjacent categories of an ORS. For ORS with *I *≥ 3, NUA models are defined by:(3)

For this model, DDs are written as:(4)

illustrating the possible DDs variations between categories, even between adjacent ones. NUA models are a generalization of UA models. Indeed, UA model is a particular case of a NUA model where parameters *β_k_*, _*k*+1 _are all equal (do not depend on *k*). Comparison of log-likelihood of data when using UA and NUA models allows us to test DDs homogeneity between adjacent categories and can provide useful information on scale structure. See Valet *et al. *[12,16] for a complete description of the NUA models and the possible patterns of distinguishability that can be tested.

### Power estimation of tests in NUA models

To investigate the ability of NUA models to detect heterogeneities within the DDs between adjacent categories, a simple method was proposed to simulate ordered contingency tables resulting from the use of ORS having different patterns of distinguishability between their adjacent categories. Hereafter, tests were defined for a null hypothesis *H*_0 _corresponding to the UA model defined by equation (2), and alternative hypotheses *H*_1 _corresponding to NUA models defined by equation (3). Different scenarios of DDs heterogeneity were proposed to illustrate different typical scale structures. In all situations, marginal homogeneity between readers was assumed, which can be expressed as: .

#### Simulation of I × I contingency tables from the NUA models

The total sample size *N *was fixed, but the row and column totals were not. Counts *n_ij _*were drawn from a full multinomial distribution *M*(*π*_*ij*_,*N*). In order to simulate different patterns of DDs heterogeneity between adjacent categories, theoretical probabilities *π*_*ij *_were defined, using equation (3), as a function of the parameters of the NUA model:(5)

When *N *and the association parameters *β*_*k, k*+1 _(*k *= 1,..., *I *- 1) are fixed, it is obvious that probabilities *π_ij _*only depend on the unknown parameters *μ *and *λ_i _*(*i *= 1,..., *I*). These *I *+ 1 unknown parameters can be defined as the solutions of the following non-linear system of *I *+ 1 equations:(6)

The first set of equations of the system defined by (6) allows us to control the marginal probabilities distribution during simulations, i.e. to control marginal probabilities . (upperscript "S" stands for simulations). The second condition of the system ensures that *μ *remains the overall effect [18]. As the number of equation is equal to the number of unknown parameters, the system can be easily solved using classical algorithm that can find roots of nonlinear systems, as the well-known Newton-Krylov method for example [19,20]. However, in this paper, a new method proposed by Lacruz *et al. *[21] was used. This "non-monotone spectral residual" method can find roots of nonlinear systems, by working without gradient information and it was shown to be competitive and frequently better than usual algorithms.

Many different scenarios of distinguishability patterns can be simulated, using different sets of {*β*_*k*,*k*+1_; *k *= 1,..., *I *- 1} in the NUA model. Suppose we aim to test all possible patterns of distinguishability, we will have to compare the null UA model (all *β*_*k, k*+1 _are equal) and NUA models with all possible combinations of association parameters, i.e. to test all possible equalities between association parameters. For example, testing equality of exactly *B *(*B *= 2,..., *I *- 1) association parameters in a NUA model with *I *- 1 association parameters would already yield to  comparisons. However, our aim was not to simulate exhaustively all possible patterns of distinguishability but credible patterns corresponding to typical scale structures in inter or intra-observer variation study. Therefore, as defined in Valet *et al. *[12] only combinations of "symmetric" and "close" association parameters were considered, that is to say NUA models where equality of some symmetric and close association parameters was assumed, respectively.

#### Definition of alternative hypotheses

For simplicity, we will consider hereafter contingency tables resulting from the use of ORS with *I *= 5 categories. The generalization to *I *× *I *contingency table is obvious. To exemplify our simulation scenarios, examples of the different values of association parameters that can be simulated in the case of a 5 × 5 contingency table, were described in table 1.

**Table 1:** Examples of association parameters and distinguishability patterns between adjacent categories from NUA models in a 5 × 5 contingency table

Hypothesis	Association parameters	Distinguishability patterns
***H*_0_**	**All association parameters are equal**	
	*β*_1,2 _= *β*_2,3 _= *β*_3,4 _= *β*_4,5 _= *log*(3)	1 ---- 2 ---- 3 ---- 4 ---- 5
	**1 association parameter is different**	
	*β*_1,2 _≠ *β*_2,3 _= *β*_3,4 _= *β*_4,5 _= *log*(3)	1 - 2 ---- 3 ---- 4 ---- 5 1-------- 2 - 3 - 4 ---- 5
	*β*_2,3 _≠ *β*_1,2 _= *β*_3,4 _= *β*_4,5 _= *log*(3)	1 ---- 2 - 3 ---- 4 ---- 5 1 -- 2-------- 3 - 4 -- 5
	*Β*_3,4 _≠ *β*_1,2 _= *β*_2,3 _= *β*_4,5 _= *log*(3)	1------ 2 -- 3 - 4 -- 5 1 -- 2 - 3 ------ 4 - 5
	*Β*_4,5 _≠ *β*_1,2 _= *β*_2,3 _= *β*_3,4 _= *log*(3)	1 ---- 2 ---- 3 ---- 4 - 5 1 - 2 -- 3 - 4 ---------- 5
	**2 association parameters are different**	
	*β*_1,2 _= *β*_2,3 _≠ *β*_3,4 _= *β*_4,5 _= *log*(3)	1-2 - 3------4------5 1------2------ 3-4-5
	*β*_1,2 _= *β*_4,3 _≠ *β*_2,3 _= *β*_3,4 _= *log*(3)	1--2---- 3---- 4--5 1 ---- 2 - 3 - 4 ---- 5
	**All association parameters are different**	
	*β*_1,2 _≠ *β*_2,3 _≠ *β*_3,4 _≠ *β*_4,5_	1 - 2 ---- 3 -- 4 ------ 5

*Distinguishabilities values between two categories are proportionnal to number of dashed-lines between these two categories

Symmetric hypotheses in association parameters:  and ,  and

From the UA model where all association parameters are equal (*H*_0 _hypothesis), a different value just for one association parameter ( hypotheses) can be used, to account for a scale defect between two categories only (categories are regularly spaced along the scale in terms of distinguishabilities, except two). Equal values for symmetric (for instance it is easier to distinguish extreme categories than to distinguish intermediate categories) or close (for instance it is easier to distinguish lower categories on the scale than upper categories) association parameters can also be used as described by hypotheses . Finally, taking different values for all association parameters ( hypothesis) illustrates an ORS where all categories are irregularly spaced in terms of distinguishabilities.

#### Distribution of marginal probabilities

In addition to the different sets of distinguishabilities values, i.e. different sets {*β*_*k*,*k*+1_; *k *= 1,..., 4} illustrating the different alternative hypotheses that can be tested, different sets of marginal probabilities  were assumed for each alternative hypothesis, to investigate the possible effects of marginal distribution heterogeneity within reader on NUA models' ability to detect significant DDs heterogeneities. These distributions were chosen in order to illustrate different realistic marginal distributions that can be observed in contingency table resulting from the classification of objects on an ORS. These different sets of marginal probabilities are described in table 2. The first set corresponds to homogeneous distribution of marginal probabilities. Then, the next three sets corresponds to homogeneous distributions except for one category with a low prevalence. The fourth and the fifth sets corresponds to homogeneous distributions except for two extreme or intermediate categories with low prevalences. The last set corresponds to an heterogeneous marginal distribution.

**Table 2:** Sets of marginal theoretical probabilities in a 5 × 5 contingency table used in our simulations

					Description
.20	.20	.20	.20	.20	Homogeneous distribution
.05	.24	.24	.24	.23	Few counts in first category
.24	.05	.24	.24	.23	Few counts in intermediate category
.24	.24	.05	.24	.23	Few counts in central category
.05	.30	.30	.30	.05	Few counts in extreme categories
.05	.05	.30	.30	.30	Few counts in the first two adjacent categories
.05	.15	.40	.30	.10	Heterogeneous distribution

#### Power and Type I error estimation

For each specific set of {*β*_*k, k*+1_; *k *= 1,..., 4} and , parameters *μ *and *λ_i _*were calculated using the non-linear system defined by (6). Probabilities *π_ij _*of the multinomial distribution were calculated from equation (5), using the specific set of {*β*_*k, k*+1_; *k *= 1,..., 4} and the previously calculated values of *μ *and *λ_i_*. Then, 10000 simulations of 5 × 5 contingency tables summarizing classifications of N objects were drawn. The same null hypothesis of equal DDs between all adjacent categories was used. For this null hypothesis, a common value *β*_1,2 _= *β*_2,3 _= *β*_3,4 _= *β*_4,5 _= log(3) was chosen, corresponding to similar association between adjacent ratings (*τ*_1,2 _= *τ*_2,3 _= *τ*_3,4 _= *τ*_4,5 _= 3) and hence similar DDs between all adjacent categories. To account for different null hypotheses, we also proposed a common value of *β*_1,2 _= *β*_2,3 _= *β*_3,4 _= *β*_4,5 _*log*(2) and *β*_1,2 _= *β*_2,3 _= *β*_3,4 _= *β*_4,5 _= *log*(4). For each simulation, the log-likelihood of UA model (*H*_0_) and NUA models defined by *H*_1 _were calculated. As proposed by several authors [12,18], the *G*^2 ^likelihood ratio-statistic was used to compare these two models. Indeed, we used the difference statistics , which are chi-squared distributed, with Δ*df *= *df_UA _*- *df_NUA _*degrees of freedom. For the different tests corresponding to hypotheses ,  and , differences Δ*df *were equal to 1, 1 and 3, respectively. For each scenario, power was estimated as the proportion of significant NUA models when applied on contincency tables simulated under the same alternative hypothesis. Type one error *α *was estimated as the proportion of significant NUA models when applied on contingency tables simulated under the null hypothesis.

## Results

**Figure 1:**
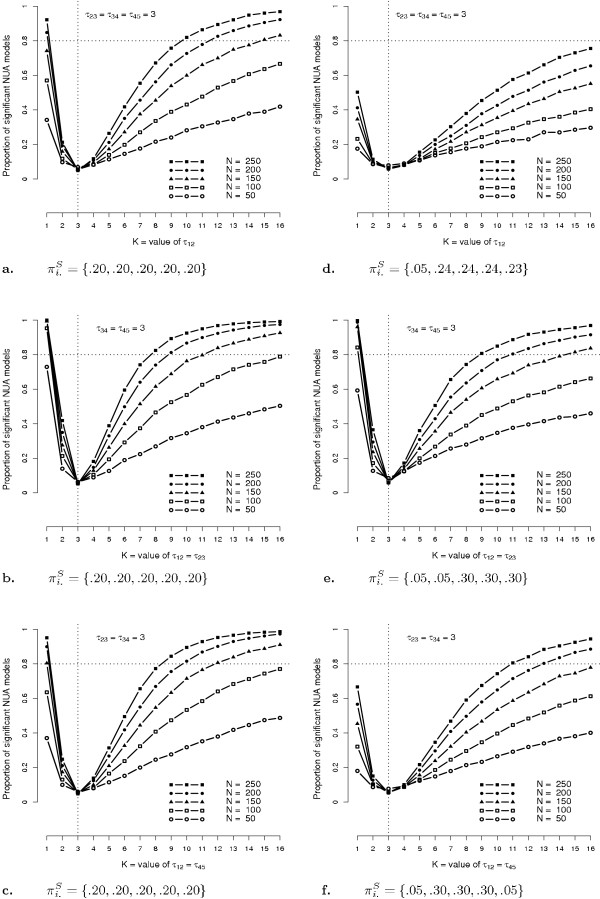
**Power estimates of tests with alternative hypotheses given by : *β*_1,2 _≠ *β*_2,3 _= *β*_3,4 _= *β*_4,5 _= *log*(3), : *β*_1,2 _= *β*_2,3 _≠ *β*_3,4 _= *β*_4,5 _= *log*(3), : *β*_1,2 _= *β*_4,5 _≠ *β*_2,3 _= *β*_3,4 _= *log*(3) for (*a, d*), (*b*, *e*) and (*c, f*) respectively**. Marginal probabilities are given by .

All simulations and power estimations were performed using R software [22]. Association parameters were equal to *log*(3) under the null hypothesis (i.e. OR equal to 3) and for each alternative hypothesis, the values *K *of the tested OR ranged from 1 to 16, which corresponds to association parameters ranging from *log*(1) = 0, to *log*(16) = 2.77. Thus, for a specific alternative hypothesis, each specific set of association parameters {*β*_*k*, *k*+1_; *k *= 1,..., 4} contained some fixed parameters equal to *log*(3) depicting the null hypothesis, and some varying parameters ranging from 0 to 2.77 depicting the alternative hypotheses. Simulations results were firstly displayed on Figure 1, illustrating for each simulated scenario, the power estimates of tests with alternative hypotheses corresponding to the different NUA models tested. In others words, this figure represents the probability of finding significant heterogeneities within the DDs between adjacent categories, according to the total sample size *N*, three different alternative hypotheses, and for different values *K *of tested OR. Left panel (Figure 1, examples a. to c.) corresponds to simulated scenarios with homogeneous marginal distributions within rater, whereas right panel (Figure 1, examples d. to f.) corresponds to simulated scenarios with three different sets of heterogeneous marginal distributions. We can observe that power estimates were constantly lower in scenarios with heterogeneous marginal distributions (right panel) as compared to those with homogeneous marginal distributions (left panel). In some cases, influence of marginal distributions heterogeneity was even drastic and strongly penalized NUA models ability in detecting significant heterogeneities within DDs between adjacent categories (Figure 1, example d.). For total sample sizes of *N *≤ 100, we can also note that none of the simulated scenarios provided power estimates greater than 80%. Conversely, except for example given in Figure 1, example d., power estimates were greater than 80% for tested OR *K *≥ 12, for all the tested hypotheses. Then, power estimates were given in table 3. Like in Figure 1, this table shows power estimates as a function of *N*, the three different alternative hypotheses, and the different values *K *of the tested OR. In a similar way, left panel corresponds to simulated scenarios with homogeneous marginal distribution, whereas right panel corresponds to different situations of heterogeneity within marginal distributions. For example, from the null hypothesis that all OR are equal to 3, i.e. DDs between all adjacent categories equal to 2/3, the power estimates of test corresponding to *i*) an alternative given by : *β*_1,2 _≠ *β*_2,3 _= *β*_3,4 _= *β*_4,5_, *ii*) an homogeneous marginal distribution, and *iii*) a total sample size equal to N = 250, are greater than 80% for OR greater or equal to 10. In others words, for N = 250, NUA models are able to detect with a probability greater than 80%, DD between adjacent categories 1 and 2, greater than 1-1/10=.90. For the left panel of this table and for the  hypothesis of a different DD between the first two adjacent categories as compared to the others, NUA models are able to detect with a probability greater than 80%: a null DD or DDs greater than .92 for *N *≥ 200, and DDs greater than .94 for *N *≥ 150. In a similar way, for *N *= 200, NUA models are able to detect different DD between close and symmetric adjacent categories ( and , respectively) with a probability greater than 80% for null DD or DDs greater than .90.

It is clear that table 3 does not provide power estimates for all possible values of association parameters tested and hence for all decimal values between *K *= 0 and *K *= 2.77. However, interpolation of power estimate for a specific value of association parameter is straightforward. From table 3 suppose for example that we want to calculate the required sample size for a common value *β*_1,2 _= *β*_2,3 _= 2.25. From power estimates corresponding to *β*_1,2 _= *β*_2,3 _= 2.20 (namely .32, .53, .69, .81 and .89) and those corresponding to *β*_1,2 _= *β*_2,3 _= 2.30 (namely .35, .57, .76, .87 and .92), we can interpolate those corresponding to 2.25 = 2.20 + (2.30 - 2.20)/2 as (0.32 + (0.35 - 0.32)/2,..., 0.89 + (.92 - .89)/2. The corresponding new values are then equal to .34, .55, .73, .84 and .91 respectively for *N *equal to 50, 100, 150, 200 and 250. Then, for a probability equal to .80, that is to say between .73 (*N *= 150) and .84 (*N *= 200), the required sample size can be interpolated as *N *= 150 + (200 - 150)/*C*, where *C *can be calculated from the following equation: 0.80 = 0.73 + (0.84 - 0.73)/*C*. For this example N has to be greater than 182.61, that is to say greater or equal to 183.

**Table 3:** Power estimates of tests in a 5 × 5 table, as a function of *N*, with three different alternative hypotheseses , with homogeneous (left column) and heterogeneous (right column) marginal theoretical distributions described by . Estimates greater than 80% are in bold

			*N*=	50	100	150	200	250		50	100	150	200	250


*β*_12_	OR	DD	**a**.						**d**.					

.00	1	.00		.34	.57	.74	**.85**	**.92**		.21	.30	.43	.54	.63
.69	2	.50		.10	.12	.16	.19	.21		.10	.10	.10	.11	.13
1.10	3	.67		.07	.06	.06	.05	.05		.09	.10	.07	.06	.06
1.39	4	.75		.08	.08	.10	.11	.12		.11	.13	.11	.10	.10
1.61	5	.80		.11	.14	.17	.21	.26		.14	.17	.16	.16	.18
1.79	6	.83		.15	.20	.27	.35	.42		.15	.21	.21	.23	.25
1.95	7	.86		.18	.27	.38	.46	.55		.17	.25	.26	.29	.33
2.08	8	.87		.22	.34	.45	.56	.67		.18	.29	.31	.34	.40
2.20	9	.88		.24	.39	.54	.66	.76		.20	.30	.35	.40	.48
2.30	10	.90		.28	.43	.60	.73	**.82**		.21	.35	.40	.44	.52
2.48	12	.92		.33	.53	.70	**.83**	**.89**		.23	.39	.45	.52	.61
2.64	14	.93		.38	.61	.78	**.89**	**.95**		.26	.43	.52	.58	.67
2.77	16	.94		.42	.67	**.83**	**.92**	**.97**		.26	.47	.56	.63	.73



*β*_12_, *β*_23_	OR	DD	**b**.						**e**.					

.00	1	.00		.73	**.95**	**.99**	**1**	**1**		.59	**.84**	**.96**	**.99**	**1**
.69	2	.50		.14	.21	.28	.35	.42		.13	.17	.24	.29	.37
1.10	3	.67		.06	.06	.05	.05	.05		.09	.07	.06	.06	.06
1.39	4	.75		.09	.10	.13	.15	.18		.12	.13	.14	.15	.17
1.61	5	.80		.13	.19	.26	.33	.39		.17	.20	.26	.31	.36
1.79	6	.83		.19	.29	.40	.50	.59		.21	.27	.36	.43	.51
1.95	7	.86		.22	.37	.51	.64	.74		.26	.34	.46	.55	.66
2.08	8	.87		.27	.47	.62	.74	**.82**		.28	.39	.54	.64	.74
2.20	9	.88		.32	.53	.69	**.81**	**.89**		.32	.45	.61	.71	.**81**
2.30	10	.90		.35	.57	.76	**.87**	**.92**		.35	.49	.66	.77	**.85**
2.48	12	.92		.41	.67	**.84**	**.92**	**.97**		.40	.56	.74	**.84**	**.92**
2.64	14	.93		.46	.74	**.89**	**.96**	**.99**		.44	.61	.79	**.88**	**.95**
2.77	16	.94		.50	.79	**.93**	**.97**	**.99**		.46	.66	**.84**	**.92**	**.97**



*β*_12_, *β*_45_	OR	DD	**c**.						**f**.					

.00	1	.00		.37	.64	**.80**	**.90**	**.95**		.18	.32	.45	.57	.67
.69	2	.50		.10	.13	.17	.21	.25		.09	.10	.11	.13	.15
1.10	3	.67		.06	.06	.05	.05	.05		.08	.06	.06	.05	.05
1.39	4	.75		.08	.08	.10	.12	.14		.09	.09	.09	.09	.10
1.61	5	.80		.11	.16	.21	.27	.31		.12	.13	.16	.18	.22
1.79	6	.83		.15	.24	.33	.42	.49		.15	.19	.24	.30	.35
1.95	7	.86		.20	.32	.44	.55	.66		.18	.25	.32	.41	.47
2.08	8	.87		.25	.41	.55	.67	.77		.21	.30	.40	.50	.59
2.20	9	.88		.28	.47	.63	.76	**.84**		.23	.35	.47	.58	.67
2.30	10	.90		.32	.53	.71	**.82**	**.90**		.27	.40	.53	.65	.74
2.48	12	.92		.38	.64	**.81**	**.90**	**.95**		.32	.18	.64	.76	**.84**
2.64	14	.93		.45	.71	**.87**	**.95**	**.98**		.37	.56	.73	**.84**	**.90**
2.77	16	.94		.49	.77	**.91**	**.97**	**.99**		.40	.61	.78	**.89**	**.94**

In a similar way, tables 4 and 5 provided power estimates for the same three different alternative hypotheses, considering at this time the null hypotheses that all OR are equal to 2 and 4, respectively. These tables allow the reader to estimate power for different null hypotheses through interpolation. Supplementary tables were also proposed to account for 4 × 4 (Additional file 1: table S1) and 6 × 6 (Additional file 1: table S2) contingency tables. In addition, results for different alternative hypotheses as well as different scenarios and sample sizes can be easily provided on simple request to the authors.

**Table 4:** Power estimates of tests in a 5 × 5 table, as a function of *N*, with three different alternative hypotheseses , with homogeneous (left column) and heterogeneous (right column) marginal theoretical distributions described by . Estimates greater than 80% are in bold

			*N*=	50	100	150	200	250		50	100	150	200	250


*β*_12_	OR	DD	**a**.						**d**.					

.00	1	.00		.22	.38	.51	.59	.72		.14	.16	.21	.26	.32
.69	2	.50		.06	.05	.05	.05	.06		.07	.07	.07	.06	.06
1.10	3	.67		.11	.13	.17	.22	.26		.10	.10	.10	.10	.14
1.39	4	.75		.16	.25	.41	.48	.57		.13	.15	.21	.25	.28
1.61	5	.80		.26	.41	.56	.67	.79		.17	.22	.29	.36	.43
1.79	6	.83		.33	.52	.70	**.82**	**.88**		.22	.30	.39	.47	.55
1.95	7	.86		.38	.63	.78	**.92**	**.94**		.25	.34	.46	.57	.66
2.08	8	.87		.43	.72	**.85**	**.94**	**.98**		.30	.41	.56	.62	.74
2.20	9	.88		.47	.76	**.90**	**.97**	**.99**		.34	.43	.58	.70	.78
2.30	10	.90		.52	.79	**.94**	**.98**	**.99**		.38	.51	.67	.74	**.86**
2.48	12	.92		.58	**.85**	**.96**	**.99**	**1**		.39	.55	.71	**.81**	**.91**
2.64	14	.93		.64	**.90**	**.97**	**1**	**1**		.41	.58	.78	**.86**	**.93**
2.77	16	.94		.69	**.95**	**.99**	**1**	**1**		.46	.62	**.84**	**.88**	**.97**



*β*_12_, *β*_23_	OR	DD	**b**.						**e**.					

.00	1	.00		.43	.74	**.87**	**.96**	**.97**		.34	.52	.73	**.86**	**.92**
.69	2	.50		.06	.06	.05	.05	.06		.08	.07	.06	.06	.05
1.10	3	.67		.12	.20	.28	.37	.44		.16	.19	.28	.35	.40
1.39	4	.75		.24	.41	.57	.66	.78		.28	.41	.54	.62	.74
1.61	5	.80		.34	.57	.78	**.86**	**.93**		.36	.52	.71	**.81**	**.89**
1.79	6	.83		.42	.69	**.86**	**.95**	**.97**		.40	.62	**.81**	**.89**	**.94**
1.95	7	.86		.51	.78	**.90**	**.97**	**.98**		.48	.71	**.89**	**.94**	**.98**
2.08	8	.87		.53	**.85**	**.95**	**.98**	**1**		.54	.76	**.93**	**.96**	**.99**
2.20	9	.88		.62	**.88**	**.97**	**1**	**1**		.55	**.80**	**.94**	**.97**	**.99**
2.30	10	.90		.64	**.90**	**.97**	**1**	**1**		.57	**.85**	**.96**	**.98**	**1**
2.48	12	.92		.69	**.93**	**.99**	**1**	**1**		.65	**.86**	**.98**	**.99**	**1**
2.64	14	.93		.73	**.96**	**.99**	**1**	**1**		.67	**.87**	**.97**	**1**	**1**
2.77	16	.94		.77	**.98**	**.99**	**1**	**1**		.71	**.93**	**.99**	**1**	**1**



*β*_12_, *β*_45_	OR	DD	**c**.						**f**.					

.00	1	.00		.20	.37	.52	.63	.74		.10	.16	.27	.32	.37
.69	2	.50		.07	.05	.05	.05	.04		.06	.07	.05	.05	.05
1.10	3	.67		.11	.12	.16	.23	.26		.10	.10	.13	.14	.18
1.39	4	.75		.19	.32	.44	.52	.61		.15	.21	.28	.34	.38
1.61	5	.80		.26	.46	.62	.74	**.82**		.21	.32	.42	.52	.60
1.79	6	.83		.35	.58	.75	**.88**	**.94**		.25	.42	.54	.71	.77
1.95	7	.86		.45	.68	**.85**	**.93**	**.97**		.31	.49	.65	.79	**.84**
2.08	8	.87		.44	.74	**.92**	**.96**	**1**		.36	.55	.73	**.86**	**.91**
2.20	9	.88		.55	**.84**	**.94**	**.98**	**.99**		.41	.61	.78	**.89**	**.94**
2.30	10	.90		.58	**.87**	**.96**	**.99**	**1**		.47	.67	**.85**	**.92**	**.96**
2.48	12	.92		.66	**.91**	**.99**	**.99**	**1**		.52	.77	**.91**	**.97**	**.98**
2.64	14	.93		.70	**.94**	**.98**	**1**	**1**		.53	**.82**	**.93**	**.98**	**1**
2.77	16	.94		.74	**.95**	**.99**	**1**	**1**		.58	**.85**	**.96**	**.98**	**1**

**Table 5:** Power estimates of tests in a 5 × 5 table, as a function of *N*, with three different alternative hypotheseses , with homogeneous (left column) and heterogeneous (right column) marginal theoretical distributions described by . Estimates greater than 80% are in bold

			*N*=	50	100	150	200	250		50	100	150	200	250


*β*_12_	OR	DD	**a**.						**d**.					

.00	1	.00		.40	.66	**.82**	**.92**	**.97**		.21	.31	.43	.50	.65
.69	2	.50		.16	.22	.31	.35	.45		.11	.12	.16	.18	.24
1.10	3	.67		.07	.09	.08	.12	.12		.09	.08	.07	.08	.08
1.39	4	.75		.06	.05	.05	.04	.06		.08	.07	.07	.06	.05
1.61	5	.80		.08	.06	.06	.08	.08		.08	.08	.08	.07	.06
1.79	6	.83		.09	.10	.12	.14	.16		.07	.09	.10	.10	.11
1.95	7	.86		.11	.11	.17	.22	.25		.12	.12	.12	.13	.15
2.08	8	.87		.13	.17	.24	.31	.36		.11	.14	.15	.18	.23
2.20	9	.88		.15	.21	.30	.37	.45		.13	.17	.20	.23	.27
2.30	10	.90		.18	.24	.34	.43	.52		.14	.19	.25	.24	.30
2.48	12	.92		.21	.35	.45	.58	.67		.19	.21	.31	.32	.40
2.64	14	.93		.24	.38	.53	.66	.77		.19	.26	.33	.42	.49
2.77	16	.94		.29	.46	.61	.76	**.84**		.20	.28	.39	.45	.55



*β*_12_, *β*_23_	OR	DD	**b**.						**e**.					

.00	1	.00		**.85**	**.99**	**1**	**1**	**1**		.71	**.92**	**.99**	**1**	**1**
.69	2	.50		.23	.43	.60	.67	.76		.22	.34	.50	.61	.72
1.10	3	.67		.09	.12	.13	.15	.18		.11	.10	.11	.11	.15
1.39	4	.75		.05	.05	.05	.06	.05		.07	.06	.08	.06	.05
1.61	5	.80		.07	.08	.11	.10	.10		.11	.08	.10	.10	.11
1.79	6	.83		.11	.11	.15	.21	.22		.16	.13	.17	.17	.20
1.95	7	.86		.14	.18	.25	.28	.35		.16	.19	.21	.29	.30
2.08	8	.87		.14	.22	.31	.40	.45		.18	.23	.27	.30	.42
2.20	9	.88		.17	.29	.41	.50	.57		.23	.26	.36	.39	.49
2.30	10	.90		.23	.33	.49	.56	.69		.23	.30	.40	.46	.53
2.48	12	.92		.25	.41	.59	.73	**.81**		.28	.33	.49	.57	.67
2.64	14	.93		.29	.51	.67	.79	**.86**		.32	.42	.55	.65	.76
2.77	16	.94		.30	.56	.75	**.86**	**.92**		.35	.47	.60	.71	**.81**



*β*_12_, *β*_45_	OR	DD	**c**.						**f**.					

.00	1	.00		.45	.77	**.90**	**.97**	**.99**		.26	.47	.60	.71	**.82**
.69	2	.50		.14	.26	.36	.44	.50		.11	.15	.21	.25	.32
1.10	3	.67		.08	.09	.10	.13	.12		.08	.09	.08	.09	.10
1.39	4	.75		.05	.06	.05	.05	.06		.08	.06	.06	.06	.06
1.61	5	.80		.06	.07	.08	.07	.09		.10	.08	.09	.08	.07
1.79	6	.83		.08	.12	.14	.16	.21		.12	.11	.12	.14	.14
1.95	7	.86		.12	.15	.20	.24	.33		.14	.14	.17	.19	.23
2.08	8	.87		.13	.22	.28	.38	.44		.14	.17	.22	.28	.33
2.20	9	.88		.20	.25	.36	.47	.56		.17	.20	.27	.35	.41
2.30	10	.90		.20	.31	.46	.55	.66		.17	.22	.33	.41	.49
2.48	12	.92		.26	.38	.54	.67	**.80**		.23	.31	.43	.50	.60
2.64	14	.93		.28	.50	.64	.77	**.87**		.26	.42	.51	.63	.73
2.77	16	.94		.35	.55	.75	**.87**	**.90**		.29	.45	.61	.70	.79

## Discussion

Results given by Figure 1 andtables 3 to 5 highlighted the strong influence that marginal heterogeneity within reader may have on power estimates of tests in NUA models. Conversely, when assuming marginal homogeneity within reader, NUA models are able to detect, from a null hypothesis of a DD equal to 2/3 between all adjacent categories and for a reasonable value of *N *= 200, null DD (between two or three categories with a probability greater than 80%. For a five-level scale, with an equal DD of 2/3 between its adjacent categories, NUA models are hence able to detect two or more confusing categories with a satisfying power. In the same way, for *N *= 200, NUA models are able to detect with a good power two or more adjacent categories (close or symmetric) for which the DDs are greater or equal to .92.

In our simulations of contingency tables resulting from cross-classifications of the same objects twice on an ordinal rating scale, the assumption of marginal homogeneity between readers was assumed, which can be seen as a limiting constraint. However, as described by the authors [12,16], NUA models are based on the assumption that in agreement studies, high values of counts are expected on the diagonal of the contingency table, and on the parallels immediately over and below this diagonal, whereas low values of counts are expected in others parts of this contingency table. Thus defined, NUA models are suitable for contingency tables with marginal homogeneities and may not be adapted for contingency tables showing others patterns of marginal distribution. In addition, it should be noticed that such patterns of contingency tables usually show a baseline non null association between adjacent ratings, what may consolidate the choice of OR = 3 under the null hypothesis.

For each simulations, the algorithm of Lacruz *et al. *[21] was used to estimate parameters *μ *and *λ_i_*. Like many others systems, this system of non-linear equations appeared to be very sensitive to initial values. In order to handle this problem and to avoid local maximums, solutions *μ *and *λ_i _*of each system associated to a specific value *K *of the tested OR were used as initial parameters of the following system with the next tested *K *value.

In this simulation study we presented three alternative hypotheses illustrating different patterns of distinguishability between adjacent categories. The first tested hypothesis  (DD between categories 1 and 2 different from the others), the corresponding symmetric hypothesis (DD between categories 4 and 5 different from the others), and the last hypothesis  (DDs between extreme adjacent categories different from the others) allow to detect significant differences between extreme adjacent categories (1 and 2, 4 and 5 or both) and others intermediate ones. This is a usual pattern in ordinal rating scales, as the first category often corresponds to "no intensity" and the last one often corresponds to the "highest intensity" of the measured phenomenon. These two extreme adjacent categories are more likely to be distinguishable than the others because they correspond to extreme situations. Finally, the second hypothesis  (DDs between close adjacent categories from 1 to 3 different then the others) and the corresponding symmetric one (DDs between close adjacent categories from 3 to 5 different from the others) allow to detect higher or lower DDs between some close adjacent categories of the scale. This can also be a typical pattern corresponding for example to ordinal scale where some consecutive grades shows many similarities and may be hard to distinguish.

## Conclusions

In this paper we proposed a new simple method based on simulations, to estimate power of tests in log-linear non-uniform association models. To this aim, we first presented a method to simulate contingency tables resulting from cross-classifications of the same objects, using ordinal rating scales having different patterns of distinguishability between their adjacent categories. Then, taking typical situations of scale structures, we proposed a table summarizing the main effects of sample size, alternative hypotheses and marginal distributions on power estimates for the detection of DDs heterogeneities within the scale structure. Results were given for three typical alternative hypotheses, and in the case of an 5 × 5 contingency tables.

In health-research assessment of disease severity or patients' well being are more and more performed using ordinal rating scales. One of the major component of an ordinal scale is category distinguishability between its adjacent categories. Using a simple method based on simulations, this paper provided some issues about how many objects has to be classified by two observers to be able to detect a given scale structure defect, what may be of prime interest to improve ordinal scale quality and then others assessments made using this scale.

## Competing interests

The authors declare that they have no competing interests

## Authors' contributions

FV and JYM developed the method, performed all statistical analyses and participated to article writing. FV and JYM read and approved the final manuscript.

## Pre-publication history

The pre-publication history for this paper can be accessed here:

http://www.biomedcentral.com/1471-2288/11/70/prepub

## Supplementary Material

Additional file 1**Power estimates of tests in a 4 × 4 table, as a function of *N*, with three different alternative hypotheseses , with homogeneous (left column) and heterogeneous (right column) marginal theoretical distributions described by {}.** Estimates greater than 80% are in bold. This table provided in the case of 4 × 4 contingency tables, power estimates, with three different alternative hypotheses and considering homogeneous (left column) and heterogeneous (right column) marginal theoretical distributions.Click here for file
