# Supplementary material for: Power estimation of tests in log-linear non-uniform association models for ordinal agreement
Source: BMC Med Res Methodol. 2011 May 17;11:70. doi: 10.1186/1471-2288-11-70 (PMC3118948; doi:10.1186/1471-2288-11-70)
Supplement: Additional file 1 — Power estimates of tests in a 4 × 4 table, as a function of N, with three different alternative hypotheseses , with homogeneous (left column) and heterogeneous (right column) marginal theoretical distributions described by {}. Estimates greater than 80% are in bold. This table provided in the case of 4 × 4 contingency tables, power estimates, with three different alternative hypotheses and considering homogeneous (left column) and heterogeneous (right column) marginal theoretical distributions. [file 1471-2288-11-70-S1.PDF]

Table s1 - Power estimates of tests in a 4×4 table, as a function of N, with three different alternative hypotheseses (H_1^{11}, H_1^{21}, and H_1^{22}), with homogeneous (left column) and heterogeneous (right column) marginal theoretical distributions described by $\{\pi_{i.}^S\}$. Estimates greater than 80% are in bold

			$N=$	50	100	150	200	250		50	100	150	200	250
H_1^{11}														
β_{12}	OR	DD	**a.**	$\{\pi_{i.}^S\} = \{.25, .25, .25, .25\}$					**d.**	$\{\pi_{i.}^S\} = \{.05, .32, .32, .31\}$				
.00	1	.00		.34	.57	.73	**.85**	**.93**		.14	.19	.31	.37	.47
.69	2	.50		.11	.13	.15	.19	.21		.07	.08	.09	.10	.14
1.10	3	.67		.06	.06	.05	.06	.05		.07	.08	.05	.05	.05
1.39	4	.75		.07	.08	.10	.10	.12		.10	.08	.09	.08	.09
1.61	5	.80		.10	.13	.20	.22	.26		.11	.10	.12	.12	.15
1.79	6	.83		.15	.20	.28	.35	.45		.15	.14	.16	.20	.23
1.95	7	.86		.17	.28	.40	.47	.59		.15	.17	.23	.24	.32
2.08	8	.87		.20	.34	.51	.58	.70		.18	.22	.28	.29	.42
2.20	9	.88		.23	.43	.56	.68	.77		.18	.26	.33	.41	.47
2.30	10	.90		.29	.44	.63	.74	**.85**		.20	.27	.35	.43	.52
2.48	12	.92		.33	.58	.76	**.84**	**.91**		.25	.34	.41	.54	.63
2.64	14	.93		.38	.65	**.81**	**.91**	**.96**		.26	.36	.53	.61	.67
2.77	16	.94		.42	.71	**.86**	**.93**	**.97**		.31	.41	.59	.66	.78
H_1^{21}														
β_{12}, β_{23}	OR	DD	**b.**	$\{\pi_{i.}^S\} = \{.25, .25, .25, .25\}$					**e.**	$\{\pi_{i.}^S\} = \{.05, .05, .45, .45\}$				
.00	1	.00		.43	.71	**.87**	**.94**	**.98**		.37	.58	.76	**.85**	**.92**
.69	2	.50		.09	.12	.18	.20	.24		.10	.10	.17	.18	.23
1.10	3	.67		.06	.06	.06	.06	.06		.09	.06	.06	.06	.04
1.39	4	.75		.08	.08	.09	.10	.14		.12	.12	.12	.12	.12
1.61	5	.80		.11	.13	.17	.21	.24		.16	.14	.20	.26	.29
1.79	6	.83		.12	.21	.24	.33	.36		.19	.20	.28	.35	.43
1.95	7	.86		.17	.22	.31	.40	.49		.22	.29	.37	.44	.54
2.08	8	.87		.19	.28	.40	.53	.61		.24	.35	.43	.53	.58
2.20	9	.88		.22	.32	.49	.59	.70		.27	.36	.50	.58	.69
2.30	10	.90		.22	.42	.57	.66	.74		.29	.41	.53	.64	.75
2.48	12	.92		.28	.49	.64	.75	**.85**		.34	.45	.64	.73	**.82**
2.64	14	.93		.33	.52	.70	**.83**	**.89**		.37	.52	.69	**.80**	**.86**
2.77	16	.94		.34	.57	.74	**.87**	**.94**		.43	.56	.72	**.84**	**.91**
H_1^{22}														
β_{12}, β_{34}	OR	DD	**c.**	$\{\pi_{i.}^S\} = \{.25, .25, .25, .25\}$					**f.**	$\{\pi_{i.}\} = \{.05, .45, .45, .05\}$				
.00	1	.00		.26	.44	.60	.72	**.82**		.08	.20	.28	.35	.44
.69	2	.50		.08	.10	.12	.15	.18		.06	.06	.08	.09	.11
1.10	3	.67		.06	.06	.05	.06	.06		.05	.05	.04	.05	.05
1.39	4	.75		.07	.09	.07	.10	.11		.08	.07	.07	.10	.09
1.61	5	.80		.09	.11	.14	.18	.23		.12	.10	.11	.13	.16
1.79	6	.83		.11	.19	.23	.31	.35		.13	.14	.18	.20	.24
1.95	7	.86		.16	.23	.32	.40	.45		.15	.18	.25	.33	.34
2.08	8	.87		.18	.28	.40	.49	.57		.15	.24	.31	.40	.46
2.20	9	.88		.17	.32	.46	.57	.69		.20	.28	.40	.46	.51
2.30	10	.90		.23	.35	.51	.64	.76		.23	.34	.43	.59	.62
2.48	12	.92		.27	.45	.63	.74	**.85**		.23	.41	.51	.65	.74
2.64	14	.93		.33	.52	.70	**.84**	**0.90**		.28	.48	.58	.73	**.80**
2.77	16	.94		.35	.59	.77	**.87**	**0.94**		.31	.53	.65	.79	**.86**

$H_1^{11} : \beta_{1,2} \neq \beta_{2,3} = \beta_{3,4} = log(3)$

$H_1^{21} : \beta_{1,2} = \beta_{2,3} \neq \beta_{3,4} = \beta_{4,5} = log(3)$

$H_1^{22} : \beta_{1,2} = \beta_{3,4} \neq \beta_{2,3} = log(3)$

Table s2 - Power estimates of tests in a 6×6 table, as a function of N, with three different alternative hypotheseses (H_1^{11}, H_1^{21}, and H_1^{22}), with homogeneous (left column) and heterogeneous (right column) marginal theoretical distributions described by $\{\pi_{i.}^S\}$. Estimates greater than 80% are in bold

			$N=$	50	100	150	200	250		50	100	150	200	250
H_1^{11}														
β_{12}	OR	DD	**a.**	$\{\pi_{i.}^S\} = \{.17, .16, .17, .16, .17, .17\}$					**d.**	$\{\pi_{i.}^S\} = \{.05, .19, .19, .19, .19, .19\}$				
.00	1	.00		.34	.53	.72	**.83**	**.89**		.18	.26	.35	.43	.53
.69	2	.50		.10	.11	.14	.17	.20		.09	.09	.11	.10	.11
1.10	3	.67		.08	.05	.04	.04	.04		.08	.08	.07	.05	.05
1.39	4	.75		.07	.10	.10	.12	.11		.08	.08	.06	.10	.08
1.61	5	.80		.12	.13	.16	.20	.24		.11	.12	.11	.12	.14
1.79	6	.83		.16	.20	.23	.33	.37		.13	.14	.16	.21	.23
1.95	7	.86		.18	.23	.34	.43	.51		.16	.18	.23	.23	.29
2.08	8	.87		.21	.31	.40	.55	.63		.17	.19	.28	.31	.38
2.20	9	.88		.23	.38	.52	.62	.69		.18	.24	.30	.38	.44
2.30	10	.90		.27	.40	.56	.70	.78		.20	.26	.34	.41	.51
2.48	12	.92		.31	.47	.64	.79	**.87**		.23	.33	.45	.51	.59
2.64	14	.93		.38	.57	.73	**.86**	**.92**		.28	.35	.50	.57	.67
2.77	16	.94		.39	.62	**.80**	**.91**	**.95**		.28	.43	.56	.67	.74
H_1^{21}														
β_{12}, β_{23}	OR	DD	**b.**	$\{\pi_{i.}^S\} = \{.17, .16, .17, .16, .17, .17\}$					**e.**	$\{\pi_{i.}^S\} = \{.05, .05, .22, .23, .22, .23\}$				
.00	1	.00		.76	**.97**	**1**	**1**	**1**		.64	**.87**	**.98**	**.99**	**1**
.69	2	.50		.16	.23	.29	.36	.45		.13	.18	.25	.30	.38
1.10	3	.67		.06	.06	.07	.05	.04		.10	.06	.06	.06	.06
1.39	4	.75		.09	.11	.13	.16	.19		.13	.10	.14	.13	.16
1.61	5	.80		.14	.20	.27	.32	.38		.19	.21	.23	.28	.36
1.79	6	.83		.17	.30	.43	.52	.59		.20	.28	.36	.43	.50
1.95	7	.86		.23	.39	.51	.67	.76		.25	.30	.45	.55	.63
2.08	8	.87		.28	.47	.64	.75	**.84**		.29	.40	.50	.62	.72
2.20	9	.88		.32	.54	.74	**.80**	**.89**		.30	.42	.60	.71	**.80**
2.30	10	.90		.36	.58	.77	**.88**	**.94**		.36	.47	.64	.75	**.85**
2.48	12	.92		.42	.69	**.86**	**.94**	**.97**		.41	.51	.74	**.81**	**.92**
2.64	14	.93		.48	.75	**.92**	**.95**	**.98**		.43	.60	.74	**.86**	**.93**
2.77	16	.94		.52	**.81**	**.93**	**.98**	**.99**		.48	.65	**.82**	**.90**	**.96**
H_1^{22}														
β_{12}, β_{56}	OR	DD	**c.**	$\{\pi_{i.}^S\} = \{.17, .16, .17, .16, .17, .17\}$					**f.**	$\{\pi_{i.}\} = \{.05, .22, .23, .22, .23, .05\}$				
.00	1	.00		.38	.68	**.83**	**.93**	**.98**		.19	.39	.49	.62	.70
.69	2	.50		.11	.17	.18	.25	.26		.10	.10	.11	.15	.16
1.10	3	.67		.07	.06	.06	.05	.06		.10	.06	.06	.07	.06
1.39	4	.75		.08	.08	.08	.12	.14		.12	.08	.09	.08	.10
1.61	5	.80		.12	.18	.22	.28	.34		.14	.14	.15	.20	.23
1.79	6	.83		.19	.26	.34	.47	.55		.15	.19	.24	.29	.33
1.95	7	.86		.21	.33	.47	.57	.71		.19	.28	.32	.42	.48
2.08	8	.87		.24	.43	.58	.70	**.80**		.20	.30	.38	.51	.56
2.20	9	.88		.27	.48	.68	.78	**.85**		.22	.36	.43	.57	.67
2.30	10	.90		.33	.54	.72	**.83**	**.91**		.26	.41	.54	.67	.74
2.48	12	.92		.37	.65	**.83**	**.92**	**.96**		.28	.48	.63	.77	**.84**
2.64	14	.93		.45	.73	**.91**	**.96**	**.98**		.34	.56	.70	**.84**	**.92**
2.77	16	.94		.50	.78	**.91**	**.98**	**.99**		.38	.64	.78	**.88**	**.93**

$H_1^{11}: \beta_{1,2} \neq \beta_{2,3} = \beta_{3,4} = \beta_{4,5} = \beta_{5,6} = log(3)$

$H_1^{21}: \beta_{1,2} = \beta_{2,3} \neq \beta_{3,4} = \beta_{4,5} = \beta_{5,6} = log(3)$

$H_1^{22}: \beta_{1,2} = \beta_{5,6} \neq \beta_{2,3} = \beta_{3,4} = \beta_{4,5} = log(3)$
